# Long-Range Corrections for Molecular Simulations with Three-Body Interactions

**DOI:** 10.1021/acs.jctc.4c01250

**Published:** 2024-12-17

**Authors:** Isabel Nitzke, Sergey V. Lishchuk, Jadran Vrabec

**Affiliations:** Thermodynamics, Technische Universität Berlin, 10587 Berlin, Germany

## Abstract

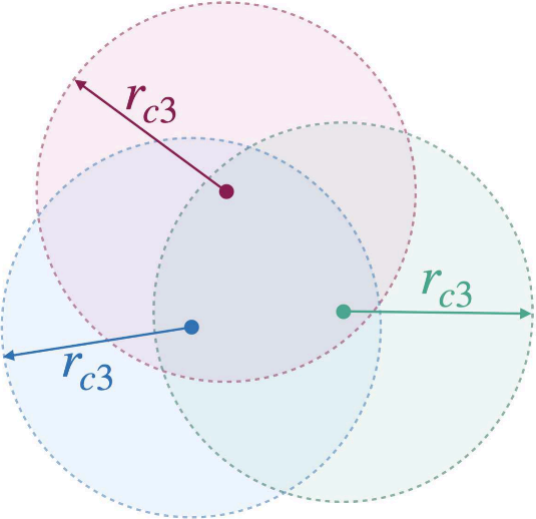

Due to their computational intensity, long-range corrections of three-body interactions are particularly desirable, while there is no consensus of how to devise a cutoff scheme. A cutoff correction scheme for three-body interactions in molecular simulations is proposed that does not rest on complex integrals and can be implemented straightforwardly. For a limited number of configurations, the three-body interactions are evaluated for a desired and a very large cutoff radius to determine the required corrections.

Molecular simulations have established themselves as an indispensable tool for science and application research. Molecular dynamics (MD) and Monte Carlo (MC) simulations provide a direct route from the interactions of molecular substances to macroscopic properties of scientific or technological interest.^1,2^ Molecular simulations rely on interaction potential models for that purpose. Pair interaction models, such as the Lennard-Jones potential, are particularly common due to their efficient evaluation with highly scalable algorithms, such as linked cell methods or Verlet lists, which enable calculations for short-range pair potentials in a linear running time *O*(*N*) based on the number of molecules *N*.

However, the limitation to pairwise additive interactions is an approximation that is only physically valid to a limited extent. The total potential energy of a real system of *N* molecules of the same species, *U*(*r*_1_, ..., *r*_*N*_), can be expanded as a series of *n*-body interactions,^3^*u*_*n*_
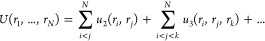
1In particular, it has been shown that nonadditive three-body interactions are essential for the quantitative description of several fluid properties, including bulk viscosity,^4^ surface tension,^5^ and speed of sound.^6^

In systems with short-range interactions, the total potential energy is dominated by interactions with neighbors close to the molecule of interest, so that it is a common practice to increase computational performance by setting the pair potential *u*_2_(*r*) to zero for intermolecular distances *r* > *r*_*c*_, where *r*_*c*_ is the specified cutoff distance. The associated error can be made arbitrarily small by choosing a sufficiently large *r*_*c*_.

As the main obstacle to taking into account many-body interactions is the extreme computational intensity, it looks natural to speed up computations by using some sort of truncation of many-body interactions similar to the cutoff distance for pair interactions. In systems of three molecules, needed to compute three-body interactions, there are three distances involved, so the obvious way of cutting off interactions would be limiting each of the three distances. Other methods can also be used to apply a cutoff, including limiting at least a pair of the distances,^7^ a sum of all three distances,^8^ or the product of all three distances.^9^ All of these approaches face some trade-off between computational efficiency and accuracy.

Systematic errors which are introduced by the truncation of pairwise intermolecular interactions are usually corrected for by adding analytic tail contributions to thermodynamic properties calculated under the assumption that the radial distribution function *g*(*r*) satisfies the condition^1,2^
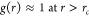
2The errors due to truncation of many-body interactions are generally lower than those for pair interactions due to faster decay of the interaction energy with the distance between molecules. There are, however, many-body contributions to the intermolecular interaction which decay relatively slowly so that computational performance would benefit from their truncation in conjunction with corresponding long-range corrections to thermodynamic properties. An example is the triple-dipole dispersion interaction derived by Axilrod and Teller,^10^ and Muto,^11^ as well as its extensions.^12^ The Axilrod–Teller–Muto (ATM) potential is given by
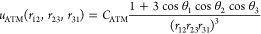
3where the *r*_*ij*_ are the lengths of the sides, θ_*i*_ are the angles of the triangle formed by three molecules, and *C*_ATM_ is the triple-dipole interaction coefficient. Being the leading term in the multipole expansion of the dispersion interaction, the ATM interaction decays more slowly with intermolecular separation than higher-order dispersion contributions as well as short-distance exchange and induced-polarization interactions. As a result, the ATM interaction is the most significant one among different long-range contributions to many-body interactions.

The long-range correction for the three-body energy can be represented by^9,13^
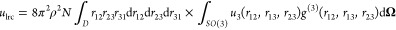
4Integration in eq 4 is carried out over sides of triangles *r*_*ij*_ = |***r***_*i*_ – ***r***_*j*_|, formed by the coordinates ***r***_*i*_ of three molecules forming a triplet, and over Euler angles **Ω** = (**ω**_1_, **ω**_2_, **ω**_3_) describing the orientations of these triangles.

Integration over orientation spans the rotation group *SO*(3) and can be carried out analytically. Integration over sides of the triangle spans the domain *D*, which depends on the three-body correction scheme. In this letter, we consider two approaches:

(i) *Pair cutoff*. This approach is a straightforward generalization of the method used for pair interaction. A three-body interaction is considered to be long-range if any of the three distances in the triplet is greater than the cutoff distance *r*_*c*2_. In this case, the domain *D* is defined by the conditions *r*_*ij*_ > *r*_*c*2_.
(ii) *Product cutoff*. The distances between the molecules in a triplet enter Equation 3 as the product *r*_12_*r*_23_*r*_31_. As pointed out by Rittger,^9^ this suggests that only the contributions from triplets with
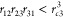
5should be computed explicitly, where *r*_*c*3_ is a constant. This condition is viewed as a three-body analogue of . In this case, the domain *D* is defined by . Explicit expressions for the integration limits are provided by Rittger.^9^

The integration domain can be defined for other approaches in a similar way.

Evaluation of the integral (4) requires knowledge of the third-order radial distribution function *g*^(3)^(*r*_12_, *r*_13_, *r*_23_). A straightforward generalization of the approach used to address this problem in the case of two-body interaction would be using the Kirkwood superposition approximation^14^
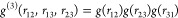
6Use of this approximation, however, is known to introduce errors in structural and thermodynamic properties.^15−17^ On the other hand, an accurate calculation of *g*^(3)^ would lead to computational overhead decreasing the benefit of using a cutoff.

In this letter, we propose a new strategy to calculate long-range corrections to thermodynamic properties in molecular simulations. The idea is to sample long-range corrections directly from simulation, within a small fraction of time steps, thus providing a universal method to reduce computational effort without additional programming effort. In the following, we illustrate the effectiveness of the proposed method with a particular case study.

For this purpose, both cutoff schemes were introduced into the simulation code *ms*2^18^ for MD. According to eq 1, a two-body potential is required to match the three-body potential. In this study, it was an augmented Tang-Toennies potential
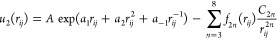
7with *f*_2*n*_ denoting the usual damping functions of the form
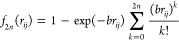
8Its parameters *A*, *a*_1_, *a*_2_, *a*_–1_, *b* and *C*_2*n*_ for *n* = 3, ..., 8, were adapted by Jäger et al. to represent krypton.^19^ The triple-dipole interaction coefficient *C*_ATM_ for the ATM potential was also taken from ref (19).

A homogeneous supercritical fluid state at 300 K was sampled with *N* = 1372 molecules in the *NVT* ensemble. Initially, the system was equilibrated for 10^4^ time steps with *r*_*c*2_ = *r*_*c*3_ = 5 σ, where σ = 3.5 Å roughly corresponds to the molecular diameter and the parameter to quantify the energy is ε, with ε/*k*_B_ = 100 K and the Boltzmann constant *k*_B_.

Upon simulation restart, a cutoff *r*_*c*3_ < *r*_*c*2_ was additionally specified. For another 10^4^ time steps, the molecular interactions were evaluated twice, i.e. up to *r*_*c*3_ = *r*_*c*2_ and additionally up to *r*_*c*3_ < *r*_*c*2_. Both results were stored, such that the long-range corrections for the residual internal energy *u* and the pressure *p* were obtained as their mean difference.

The resulting long-range corrections were validated with additional simulations where *r*_*c*3_ < *r*_*c*2_ was fixed from the beginning and used to determine the interactions. A simulation run comprised 2 × 10^5^ time steps for equilibration, followed by a production run of 10^7^ time steps. Figures 1 and 2 illustrate the behavior of *u* and *p* for various values of *r*_*c*3_ as well as the outcome when the previously obtained long-range correction was added to the new simulation results.

**Figure 1:**
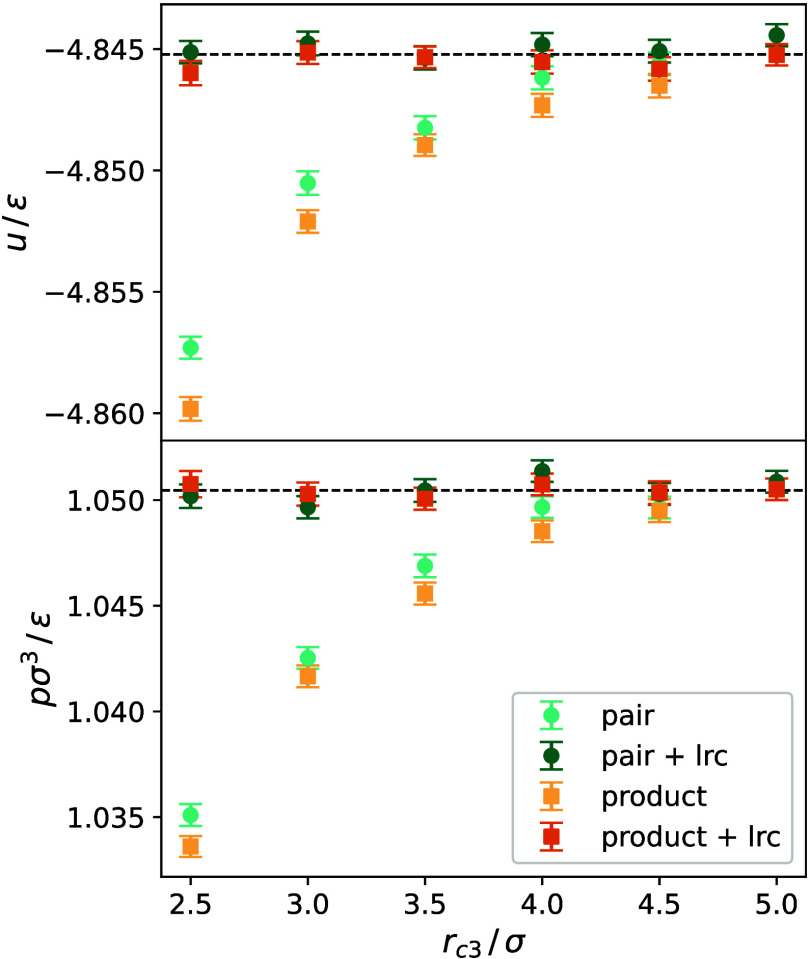
Reduced residual internal energy *u*/ε (top) and reduced pressure *p σ*^3^/ε (bottom) at *T* = 300 K and ρ = 16 mol l^–1^ for both cutoff schemes with and without long-range correction (lrc). Black dashed lines indicate the mean value.

**Figure 2:**
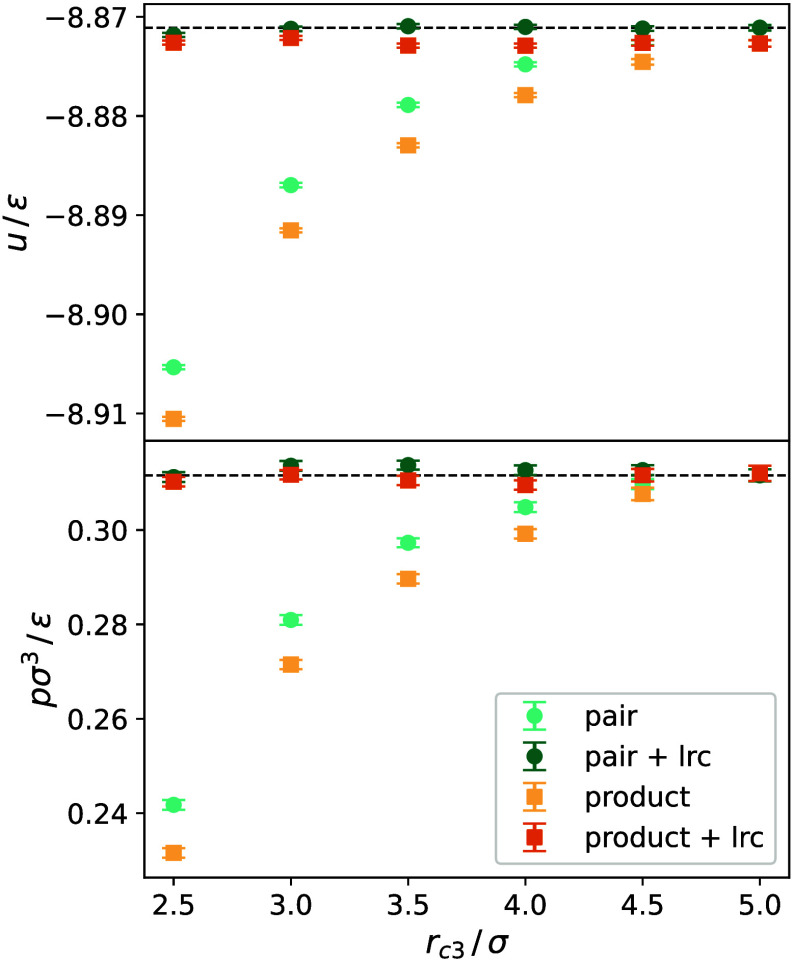
Reduced residual internal energy *u*/ε (top) and reduced pressure *p σ*^3^/ε (bottom) at *T* = 150 K and ρ = 26.76 mol l^–1^ for both cutoff schemes with and without long-range correction (lrc). Black dashed lines indicate the mean value.

Clearly, the approach to sample for a few time steps up to a large cutoff radius, while simultaneously to a chosen smaller one, and using their difference for the long-range corrections, works for both cutoff schemes.

Furthermore, it can be seen that the pairwise three-body cutoff scheme shows for a given cutoff radius slightly smaller deviations from the converged value than the product three-body cutoff scheme. A triplet satisfying the pair constraint necessarily also satisfies the product constraint, but the converse does not hold. Hence, the product scheme considers triplets that are neglected by the pair scheme.

Since cutoff schemes were developed to reduce the overall computation time in the first place, the calculation of interactions for additional triplets creates another drawback of the product scheme. Figure 3 shows the average computation time for 10^3^ MD time steps. All simulations were executed with the same MPI parallelization scheme and on the same computer architecture. A speedup of the simulation is of course achieved by any reduction of *r*_*c*3_, but less for the product cutoff scheme than for the pair cutoff scheme, which is 10–25% faster.

**Figure 3:**
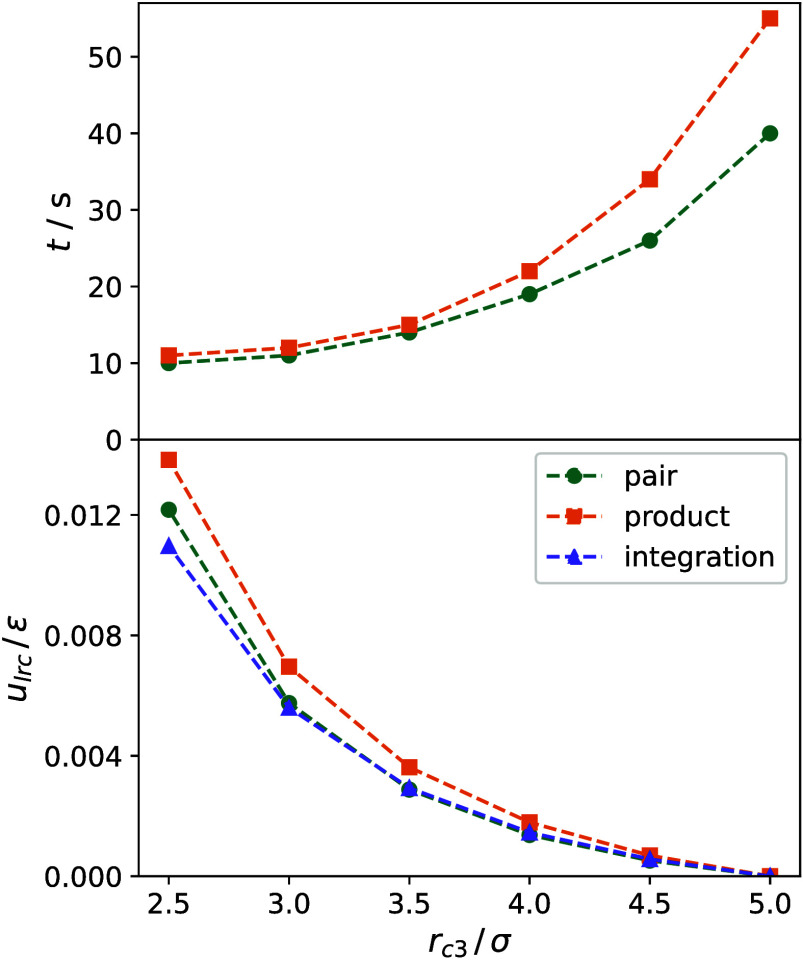
Simulation time for 1000 MD steps (top) and dependence of the long-range correction to *u*/ε obtained from simulation and integration (bottom) at *T* = 300 K and ρ = 16 mol l^–1^. Error bars are within symbol size.

The presented method for calculating long-range corrections does not involve approximations that are typically used in analytical approaches. In order to illustrate the error introduced by such approximations, we calculated the long-range correction to the three-body energy by numerically evaluating the integral in eq 4. For this purpose, the superposition approximation was employed, namely, eq 6 which avoids computation of the third-order radial distribution function and does not require additional simulations. Moreover, an assumption analogous to eq 2 was used for the pair interactions, which is necessary to avoid computation of *g*(*r*) at large distances.

Since at least two or three distances between molecules in triplets excluded by the cutoff criterion are larger than *r*_*c*3_, the approximation *g*(*r*) ≈ 1 at *r* > *r*_*c*3_ was adopted. However, this approximation cannot be used for the third (smallest) distance because it is generally arbitrary, and the integral (4) would diverge due to the large contribution from the three-body interaction at small distances. Still, the overall contribution of such triplets is small because of the repulsion due to the pair interaction. We therefore assumed that the radial distribution function for the shortest distance in the triplet is equal to the Boltzmann factor, *g*(*r*) = exp(−*u*_2_(*r*)/*k*_B_*T*), which tends to unity as *r* increases. Note that this approximation is usually used at low densities^20^ when the pair interaction between a molecule and its closest neighbor is dominant compared to interactions with other molecules in the system. In the present case, the pair interaction with the nearest neighbor is dominant over other interactions, if the molecules are very close to each other so that interactions with other molecules can be neglected. With these approximations, the third-order radial distribution function is given by
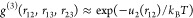
9where *r*_12_ < *r*_13_, *r*_12_ < *r*_23_, and the long-range correction for the three-body energy, eq 4, can be estimated without additional simulation data on the third-order radial distribution function *g*^(3)^.

The results of a numerical integration using a GSL implementation of the VEGAS Monte Carlo integration algorithm^21,22^ over the domain for the product cutoff approach in comparison with simulation data are shown in Figure 3. For larger cutoff radii, numerical integration and pair cutoff data show a surprising agreement, which slightly deteriorates at smaller radii *r*_*c*3_ where the simulation data are underestimated.

We have proposed a new strategy to calculate long-range corrections to thermodynamic properties in molecular simulations and illustrated the effectiveness of the proposed method with a particular case study. This strategy is universal, as it is applicable to modeling a wide range of thermodynamic properties using different methods (MD, MC) and any combination of pair and three-body interaction potentials, without additional analytical or programming effort. At the same time, it allows reducing computation effort because simulation involving the computationally expensive long-range part of the three-body interaction is carried out only in a small fraction of time steps.
